# A Case of Combined Septic and Obstructive Shock: Usefulness of Bedside Integrated Cardiothoracic Emergency Ultrasonography

**DOI:** 10.1155/2013/154861

**Published:** 2013-05-23

**Authors:** Maurizio Zanobetti, Eleonora De Villa, Delia Lazzeretti, Alberto Conti, Riccardo Pini

**Affiliations:** ^1^Intensive Observation Unit, Emergency Department, Careggi University Hospital, University of Florence, Florence, Italy; ^2^SOD Osservazione Breve Intensiva, Azienda Ospedaliero-Universitaria Careggi, Largo Brambilla 3, 50134 Firenze, Italy

## Abstract

A 59-year-old woman presented at the emergency department with cough and weakness that started a few days before. She had a history of breast cancer treated with mastectomy with negative followup. Physical examination revealed tachycardia and tachypnea, normal blood pressure, lower lobe crackles bilaterally, and jugular venous distention. Laboratory data underlined neutrophilic leukocytosis, mild renal failure, and high procalcitonin. Chest radiography revealed bilateral nodular lesions, presumably secondary. Patient was treated with fluid therapy and broad-spectrum antibiotic therapy because of suspected sepsis. In clinical revaluation patient showed systolic hypotension unresponsive to fluid resuscitation. Because of suspected pulmonary embolism an echocardiography was performed revealing normal dimensions of right ventricle with presence of a hypoechoic mass involving tricuspid annulus and obstructing the opening of anterior tricuspid flap; inferior vena cava appeared dilated and not collapsible. Subsequently, chest ultrasonography was performed, confirming multiple rounded lesions involving the pleura bilaterally, compatible with metastasis, and absence of interstitial syndrome. Finally a computed tomography scan of chest excluded pulmonary embolism and confirmed the presence of the obstructive mass responsible for hemodynamic instability together with pulmonary sepsis.

## 1. Introduction

The most frequent shock presented in emergency department (ED) [1] and in intensive care unit (ICU) [2] is the septic shock. First line treatment is fluid resuscitation. In addition to fluid resuscitation, adrenergic agents can be required to correct hypotension [3]. Bedside ultrasonography can be a useful diagnostic tool to discriminate the etiology of hypotension both in traumatic and in nontraumatic patients [1]. In fact, Focused Assessment Sonography for Trauma [4] protocol can be performed to rule out an abdominal blood loss, cardiac tamponade, and pleural effusion not only in traumatic hypotension [1–5]. Furthermore chest ultrasonography seems to be able to replace chest radiography in patients with acute dyspnea [6]. An emergency echocardiography assessment can detect a hyperdynamic left ventricular function [1, 7]. It is also a noninvasive procedure to obtain information about central venous pressure of vascular fluid status and fluid responsiveness [8, 9] through vena cava evaluation. 

## 2. Case Presentation

**Figure 1:**
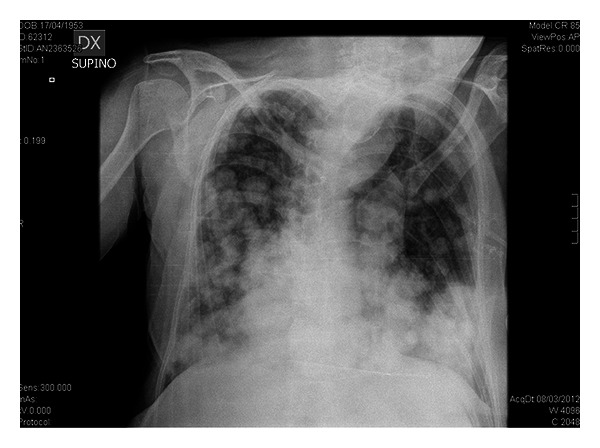
Nodular lesion at chest X-ray. Chest radiography showing bilateral nodular lesions, above all in lower lung fields.

**Figure 2:**
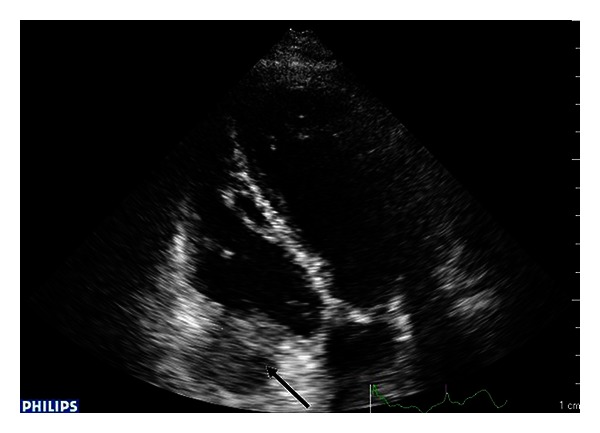
Hypoechoic mass involving tricuspid annulus. Echocardiography apical four chambers view showing a hypoechoic mass involving tricuspid annulus and right ventricle outflow tract (arrow).

**Figure 3:**
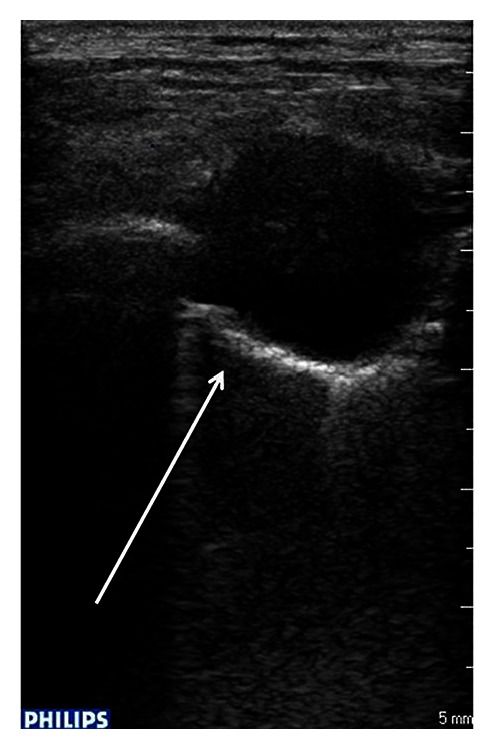
Rounded lesion involving the pleura. Chest ultrasonography showing a rounded lesion involving the pleura with rear-wall reinforcement and a localized alveolar-interstitial syndrome (arrow).

**Figure 4:**
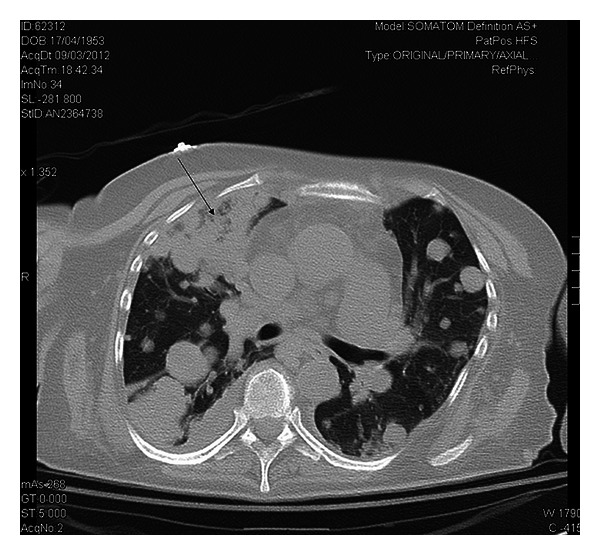
Lung abscess. Chest high-resolution computed tomography pulmonary angiography showing in the right upper lung lobe an area of consolidation with air inside a cavitary lesion (lung abscess probably) (arrow).

**Figure 5:**
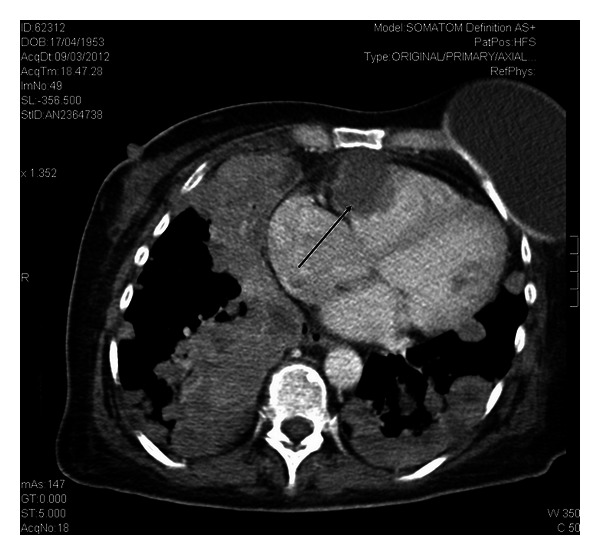
Mass involving right ventricular sections. Chest high-resolution computed tomography pulmonary angiography showing mass pertaining to the pericardium with intracardiac extension at level of anterior right ventricle wall causing occlusion of tricuspid valve (arrow).

A 59-year-old woman presented in the ED with productive cough, malaise, and progressive weakness started a few days before. She had a history of breast cancer treated with mastectomy, chemotherapy, and radiotherapy about 6 years ago. She reported regular followup until 2010. Moreover, her medical history included blood hypertension and multinodular goiter with normal thyroid function. On initial presentation the patient showed normal blood pressure, tachycardia (160 beats/min) and tachypnea (respiratory rate: 38/min), oxygen saturation 91% on room air, and body temperature 37.6°C. Chest auscultation revealed reduced breath sounds with lower lobe crackles bilaterally. Jugular venous distention was detected at physical examination. The 12-lead EKG showed atrial fibrillation with elevated mean ventricular rate (160 b/min). Arterial blood gas analysis showed hypoxia (PaO_2_ 62 mmHg) and elevated serum lactate (3.6 mmol/L). Laboratory data was significant for neutrophilic leukocytosis (WBC 19.8 × 10^9^/L with 90% neutrophils), mild renal failure (creatinine 1.48 mg/dL), elevated D dimer (1314 ug/L), and procalcitonin (1.23 ng/mL). Chest radiography revealed bilateral nodular lesions, presumably secondary, above all in lower lung fields (Figure 1). For suspected pulmonary embolism a venous duplex US scan was performed and deep venous thrombosis in lower limbs was excluded. During her staying in emergency room, the patient was treated with fluid therapy and broad-spectrum antibiotic therapy for sepsis. In clinical revaluation, the patient showed systolic hypotension unresponsive to fluid resuscitation, so she was treated with IV noradrenaline. Because of suspected pulmonary embolism an echocardiography was performed revealing normal left ventricular dimensions, normal systolic function, dilatation of left atrium, normal dimensions for both right atrium and right ventricle with presence of a hypoechoic mass involving tricuspid annulus, and right ventricle outflow tract obstructing the opening of anterior tricuspid flap (Figure 2). Moreover, inferior vena cava appeared dilated and not collapsible. Echocardiography findings revealed suspicions of obstructive shock. Subsequently, chest ultrasonography was performed, detecting a consolidation in the right lung and confirming multiple rounded lesions involving the pleura bilaterally, compatible with metastasis (Figure 3), and absence of interstitial pattern. A chest high-resolution computed tomography pulmonary angiography was performed, revealing absence of pulmonary embolism, presence of numerous nodular lesions with multiple cervical lymphadenopathies and an area of consolidation with air inside a cavitary lesion (lung abscess probably) (Figure 4) in the right upper lung lobe; moreover, a mass was detected pertaining to the pericardium with intracardiac extension at level of anterior right ventricle wall causing occlusion of tricuspid valve (Figure 5). According to the serious medical conditions, we informed relatives about the poor prognosis and the patient was transferred to a medical division, where she died the day after.

## 3. Discussion

Obstructive shock is characterized by a mechanical defect in filling and emptying of the heart and large vessels. Pulmonary embolism and cardiac tamponade are the most frequent presentations, while intracardiac tumors or thrombi are the rarest [10, 11]. In our case the patient showed at the presentation signs of severe sepsis, evolved subsequently in a suspected septic shock, so ultrasonography evaluation was aimed to evaluate fluid status and search for infection source. 

In spite of what is expected in a septic shock, inferior vena cava was dilated and without collapsibility. This finding, added to the absence of lung interstitial syndrome and normal left ventricular systolic function at the cardiothoracic evaluation, was indicative of probably obstructive shock. A detailed echocardiographic examination clarified the cause of the obstruction, showing the presence of a mass responsible for hemodynamic instability together with sepsis. 

## 4. Conclusion

Our case suggests that bedside integrated cardiothoracic emergency ultrasonography is a useful tool to identify the cause of shock in a reasonably short time in ED. 

## Abbreviations

ED: Emergency department.
